# Physical symptoms and anxiety and depression in older patients with advanced cancer in China: a network analysis

**DOI:** 10.1186/s12877-024-04788-7

**Published:** 2024-02-23

**Authors:** Mo Tang, Zhongge Su, Yi He, Ying Pang, Yuhe Zhou, Yu Wang, Yongkui Lu, Yu Jiang, Xinkun Han, Lihua Song, Liping Wang, Zimeng Li, Xiaojun Lv, Yan Wang, Juntao Yao, Xiaohong Liu, Xiaoyi Zhou, Shuangzhi He, Yening Zhang, Lili Song, Jinjiang Li, Bingmei Wang, Lili Tang

**Affiliations:** 1 Key Laboratory of Carcinogenesis and Translational Research (Ministry of Education/Beijing), Department of Psycho-oncology, Peking University Cancer Hospital & Institute, Fu-Cheng Road 52, Hai-Dian District, Beijing, 100142 China; 2 Department of Breast Cancer Radiotherapy, Chinese Academy of Medical Sciences, Cancer Hospital, Shanxi Medical University, Taiyuan, China; 3 The Fifth Department of Chemotherapy, The Affiliated Cancer Hospital of Guangxi Medical University, Guangxi Zhuang Autonomous Region, Nanning, China; 4 Department of Medical Oncology, Cancer Center, West China Hospital, Sichuan University, Chengdu, China; 5 Department of Breast Medical Oncology, Shandong Cancer Hospital and Institute, Shandong First Medical University, Shandong Academy of Medical Sciences, Jinan, China; 6 Department of Oncology, the First Affiliated Hospital of Zhengzhou University, Zhengzhou, China; 7 Department of Oncology, Xiamen Humanity Hospital, Xiamen, China; 8 Department of Integrated Chinese and Western Medicine, Shaanxi Provincial Cancer Hospital Affiliated to Medical College of Xi’an Jiaotong University, Xi’an, China; 9 Department of Clinical Spiritual Care, The Affiliated Cancer Hospital of Xiangya School of Medicine, Hunan Cancer Hospital, Central South University, Changsha, China; 10 Radiotherapy Center, Hubei Cancer Hospital, Wuhan, China; 11 Department of Psycho-oncology, Shandong Cancer Hospital and Institute, Shandong First Medical University, Shandong Academy of Medical Sciences, Jinan, China

**Keywords:** Physical symptoms, Anxiety, Depression, Older patients, Network analysis, Advanced cancer

## Abstract

**Background:**

Little is understood about the association between psychosomatic symptoms and advanced cancer among older Chinese patients.

**Methods:**

This secondary analysis was part of a multicenter cross-sectional study based on an electronic patient-reported outcome platform. Patients with advanced cancer were included between August 2019 and December 2020 in China. Participants (over 60 years) completed the MD Anderson Symptom Inventory (MDASI) and Hospital Anxiety and Depression Scale (HADS) to measure symptom burden. Network analysis was also conducted to investigate the network structure, centrality indices (strength, closeness, and betweenness) and network stability.

**Results:**

A total of 1022 patients with a mean age of 66 (60–88) years were included; 727 (71.1%) were males, and 295 (28.9%) were females. A total of 64.9% of older patients with advanced cancer had one or more symptoms, and up to 80% had anxiety and depression. The generated network indicated that the physical symptoms, anxiety and depression symptom communities were well connected with each other. Based on an evaluation of the centrality indices, ‘distress/feeling upset’ (MDASI 5) appears to be a structurally important node in all three networks, and ‘I lost interest in my own appearance’ (HADS-D4) had the lowest centrality indices. The network stability was relatively high (> 0.7).

**Conclusion:**

The symptom burden remains high in older patients with advanced cancer in China. Psychosomatic symptoms are highly interactive and often present as comorbidities. This network can be used to provide targeted interventions to optimize symptom management in older patients with advanced cancer in China.

**Trial registration:**

Chinese Clinical Trial Registry (ChiCTR1900024957), registered on 06/12/2020.

**Supplementary Information:**

The online version contains supplementary material available at 10.1186/s12877-024-04788-7.

## Introduction

In 2022, approximately 55.8% of cancer patients in China were older than 60 years of age (older people), and these patients accounted for more than 68.2% of cancer-related deaths [1]. Due to population aging and increases in life expectancy, the cancer burden among older people has been increasing, thus imposing a considerable burden on the Chinese health care system [2]. Moreover, most older cancer patients, especially those in the advanced stage, suffer from a high prevalence of frailty, declines in functional status and malnutrition, and a high rate of comorbidity [3, 4]. Older cancer patients are prone to cooccurring psychosomatic symptoms; these interrelated and synergistic symptoms are called symptom clusters [5]. Symptom clusters are closely related to cancer prognosis and quality of life [6]. Therefore, the cancer burden in older patients is of greater concern. However, older patients are underrepresented in existing cancer clinical trials and treatment guidelines, resulting in a lack of clinical evidence for symptom management in this population.

With the development of information technology and the innovation of real-world research concepts, recent advances in network analysis have provided a novel approach to deeply understanding the complex nature of symptom clusters [7]. Symptom network analysis can enable collective characterization of patients’ symptoms and quantify and visualize the correlation between different symptoms; this approach has been widely used in psychosocial oncology in recent years [8–10]. Given that cancer patients present with multiple symptoms, greater attention is warranted to understand how various symptoms can interact to improve individualized geriatric cancer management models and intervention strategies.

Previous studies have focused on the single aspect of physical [11] or psychological [12] symptom burden while ignoring their interaction. Furthermore, few relevant studies have used network analysis to focus on the symptom burden of older patients with advanced cancer in China. To this end, this study used a network analysis approach to gain insights into the complex nature of cooccurring symptoms, explore the interrelationships between the burdens of physical and psychological symptoms and identify core symptoms in Chinese geriatric advanced cancer patients using network analysis.

## Methods

### Study participants

This secondary analysis was part of a multicenter cross-sectional study based on an electronic patient-reported outcome platform [13]. The study was conducted between August 2019 and December 2020 in 10 diverse geographical and economic provinces and municipalities in China. The study process was described in detail in our previous publications [14, 15]. The inclusion criteria for the present study were as follows: (1) age ≥ 60 years; and (2) diagnosed with advanced stage lung, colorectal, gastric, liver, breast, or esophageal cancer [16]. We excluded patients with cognitive impairment and those who were unwilling or unable to fill out electronic questionnaires on their tablets or smartphone.

### Measurements

(1) The MD Anderson Symptom Inventory (MDASI) [17] is a validated assessment tool that measures 13 core symptoms in terms of severity. Respondents answer questions about aspects of daily life in the last 24 h on numeric rating scale ranging from 0-10-points, with higher scores indicating worse symptoms. Scores of 5–6 were considered to indicate moderate symptoms, and scores of 7–10 were considered to indicate severe symptoms. In this study, the severity of symptom burden was assessed by the total number of items rated as “moderate to severe” (MS). (2) The Hospital Anxiety and Depression Scale (HADS) [18] anxiety subscale (HADS-A) and depression subscale (HADS-D) were administered. Each subscale contains 7 items each that are scored on a 0–3 point scale, with higher scores indicating higher anxiety or depression levels. The scores of each subscale were categorized as follows: 0–7 indicate no depression or anxiety; 8–10 indicated mild depression or anxiety; 11–14 indicated moderate depression or anxiety; and 15–21 indicated severe depression or anxiety. The Chinese versions of the MDASI and HADS have satisfactory psychometric properties and were used in this study [19, 20].

### Statistical analysis

Descriptive statistical analysis was performed for demographic distribution and cancer characteristics of patients using SPSS 22.0. Continuous variables were expressed as the median or mean, standard deviation (SD), while categorical variables were expressed as percentages (%). Network analysis was conducted with the R ‘*bootnet*’ (version 1.4.3) [21] and ‘*qgraph*’ (version 1.6.9) [22] packages. First, we used the graphical Lasso based on an extended Bayesian information criterion option in the ‘*qgraph*’ package to construct the network structure. Second, three common node centrality indices [23], i.e., “strength, closeness, and betweenness”, were calculated using the centrality plot function of the ‘*qgraph*’ package. Third, the ‘*bootnet*’ package was used to check the sequence invariance of the nodes on the central index. The stability of the centrality index is quantified by calculating the correlation stability coefficient, which is required to be above 0.25 and preferably above 0.50 [21]. Finally, a nonparametric bootstrap procedure was used to assess the accuracy of the edge weights based on the 95% confidence intervals (CIs) determined using the ‘*bootnet*’ package. Edge accuracy was assessed by 95% CIs, with a narrower CI indicating a more trustworthy network [21].

## Results

### Participant characteristics

We included 1022 patients with a mean age of 66 (60–88) years in the present study. Specifically, 71.1% (*n* = 727) of the patients were males, and 28.9% (*n* = 295) were females. Most of the patients reported not having a college education (88.5%). The majority of patients lost ≤ 5% weight, and almost half of the patients were in relatively good physical condition. In terms of cancer types, 274 (26.8%) had lung cancer, 157 (15.4%) had colorectal cancer, 174 (17.0%) had gastric cancer, 130 (12.7%) had liver cancer, 67 (6.6%) had breast cancer, and 220 (21.5%) had esophageal cancer. For cancer response evaluation, stable disease (39.0%) made up the largest proportion, followed by progressive disease (30.1%), partial response (16.8%) and complete response (2.3%). The rest of the cancer statuses were unclear (11.6%). The baseline characteristics of the participants are included in Table 1.

**Table 1:** Baseline characteristics of the participants included in the study

Participant Characteristics	Overall (*n* = 1022)
Age (years)	66 [60–88]
Sex (n, %)	
Female	295 (28.9)
Male	727 (71.1)
Education (n, %)	
No college	904 (88.5)
College and above	118 (11.5)
Weight loss within 6 months (n, %)	
≤ 5%	706 (69.1)
5 − 10%	182 (17.8)
10 − 20%	68 (6.7)
> 20%	25 (2.4)
Unclear	41 (4.0)
Performance status, ECOG (n, %)	
0	567 (55.5)
1	294 (28.8)
2	111 (10.9)
≥ 3	50 (4.9)
Cancer response evaluation (n, %)	
Completely response	24 (2.3)
Progressive disease	308 (30.1)
Partial response	172 (16.8)
Stable disease	399 (39.0)
Unclear	119 (11.6)
Cancer types	
Lung	274 (26.8)
Colon-rectum	157 (15.4)
Stomach	174 (17.0)
Liver	130 (12.7)
Breast	67 (6.6)
Esophageal	220 (21.5)

ECOG = Eastern Cooperative Oncology Group. Functional status was evaluated on a scale from 0 to 5.

### Descriptive statistics of the measurements

**Fig. 1:**
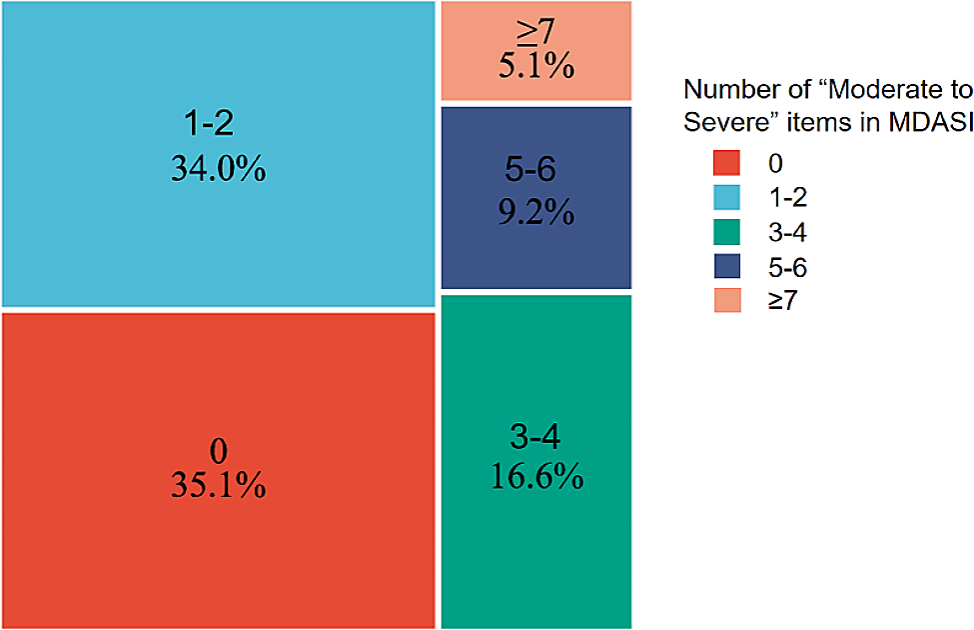
Symptom burden based on the MD Anderson Symptom Inventory

**Fig. 2:**
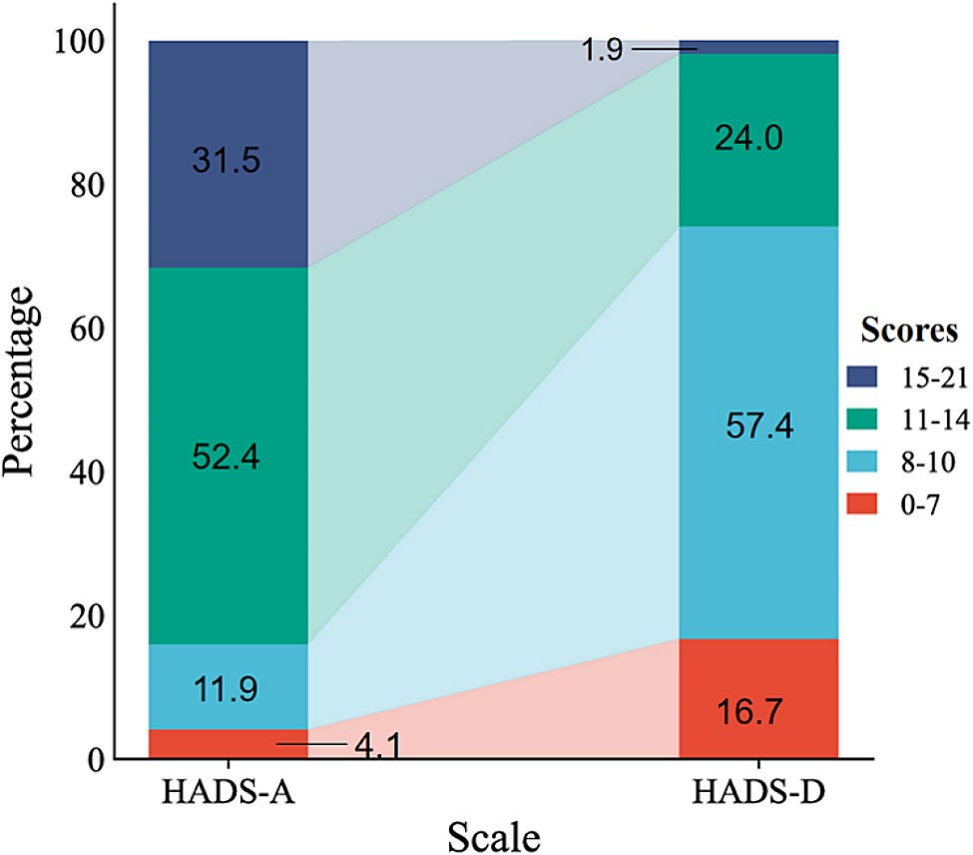
The Hospital Anxiety and Depression Scale results

As shown in Figs. 1, 2 and 34.0% of participants had one or more MS symptoms, 16.6% had more than three MS symptoms, 9.2% had more than five MS symptoms, and 5.1% had seven or more MS symptoms. Among the participants, the prevalences of anxiety and depression symptom were 95.8% and 83.3%, respectively. Of these, 83.9% had moderate to severe anxiety symptom, but only 25.9% had moderate to severe depression symptom. The mean (SD) HADS-A and HADS-D scores were 12.9 (2.6) and 9.4 (2.3), respectively.

### Network analysis

(1) Network structure

**Fig. 3:**
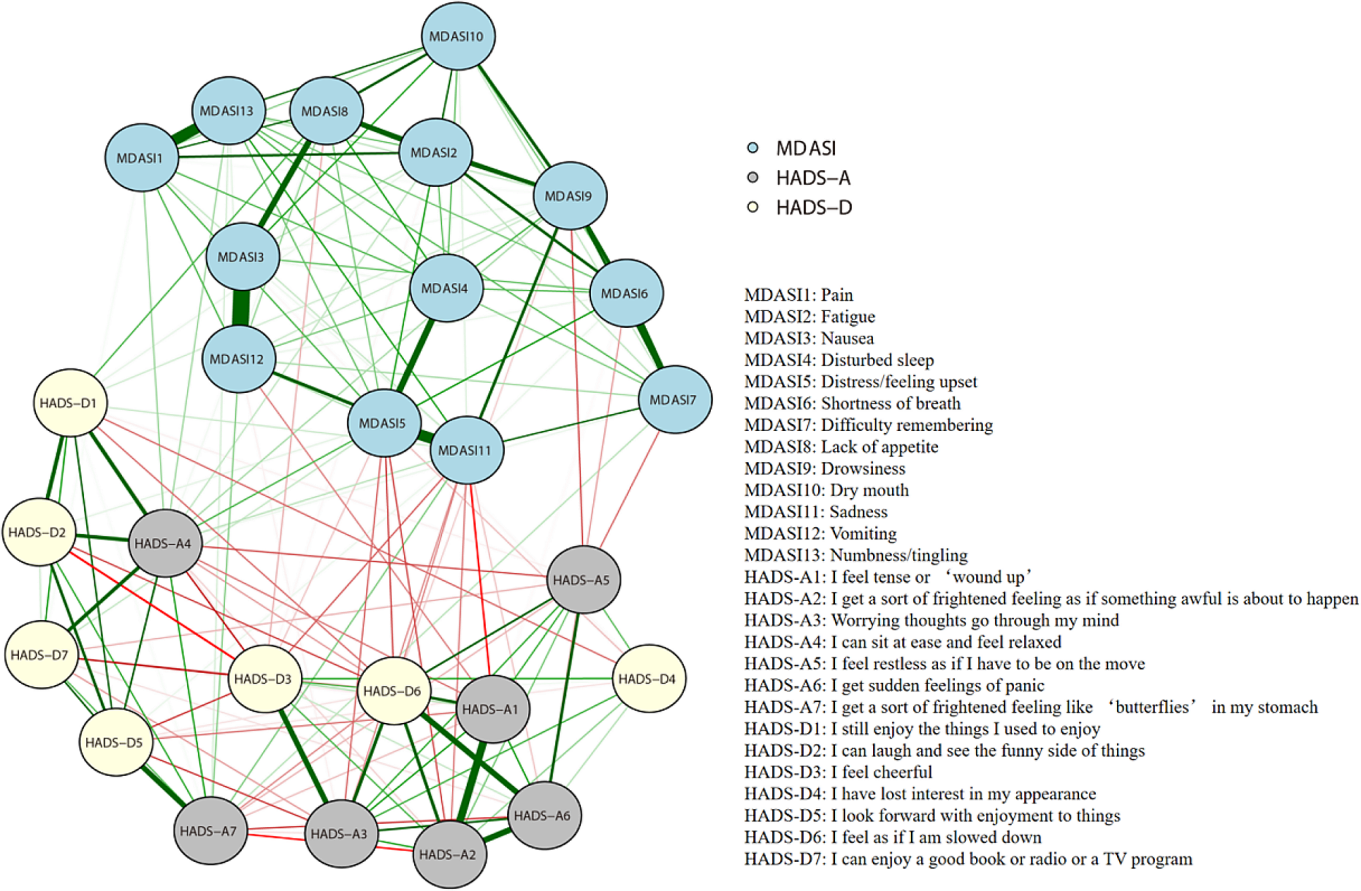
Network analysis of core symptoms, anxiety and depression. The blue lines represent positive associations, the red lines represent negative associations, and the thickness of an edge represents the strength of connectedness. The absence of edges between nodes denotes statistical independence. MDASI: MD Anderson Symptom Inventory; HADS: Hospital Anxiety and Depression Scale

The network shows the strength of the relationships between MDASI core symptoms and anxiety and depression symptoms, as shown in Fig. 3. The edges between ‘Nausea’ and ‘Vomiting’ (MDASI 3-MDASI 12, edge weight = 0.703), ‘Distress/feeling upset’ and ‘Sadness’ (MDASI 5 - MDASI 11, edge weight = 0.696), and ‘I feel tense or ‘wound up’’ and ‘I get a sort of frightened feeling as if something awful is about to happen’ (HADS-A1-HADS-A2, edge weight = 0.601) had the strongest positive edges within their respective communities. In addition, ‘Disturbed sleep’ was the most closely associated physical symptom, with ‘Distress/feeling upset’ (MDASI 4 - MDASI 5, edge weight = 0.548), and ‘Drowsiness’ was the most closely associated physical symptom with ‘Sadness’ (MDASI 9 - MDASI 11, edge weight = 0.512) (Supplementary file 1).

(2) Centrality indices

**Fig. 4:**
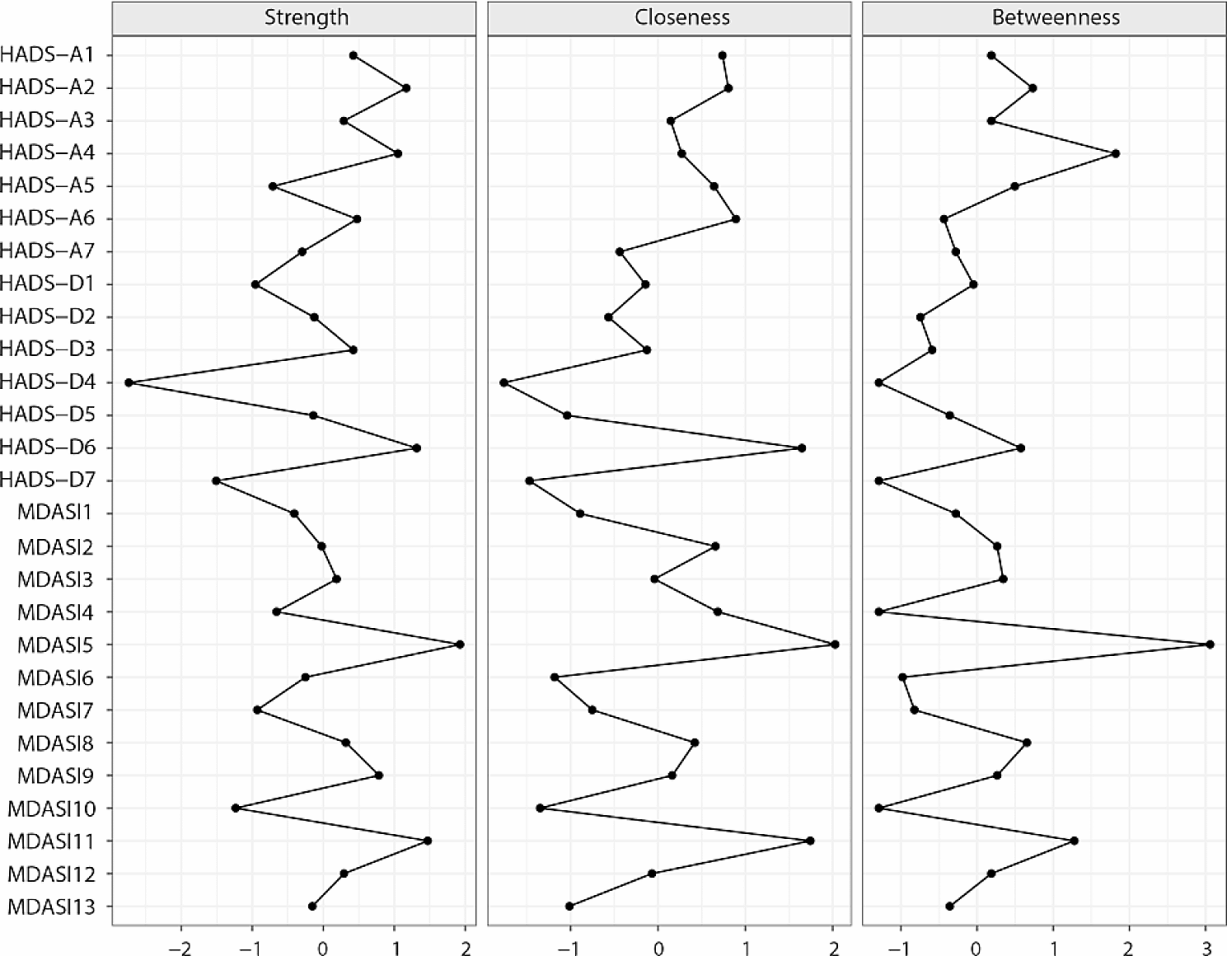
Centrality indices of node strength, closeness, and betweenness of the estimated network. The indices are shown as standardized z scores. MDASI: MD Anderson Symptom Inventory; HADS: Hospital Anxiety and Depression Scale

The strength, closeness, and betweenness centrality z scores are illustrated in Fig. 4. Overall, ‘Distress/feeling upset’ (MDASI 5) had the highest strength value, the highest closeness value and the highest betweenness value. These findings indicate that this symptom has strong connections to nearby nodes and plays an important role in the network, and its activation has the strongest influence on the other nodes in the network. Moreover, it acts as the bridge connecting the communities of nodes. ‘I lost interest in my own appearance’ (HADS-D4) had the lowest strength, closeness, and betweenness values.

(3) Stability of centrality indices

**Fig. 5:**
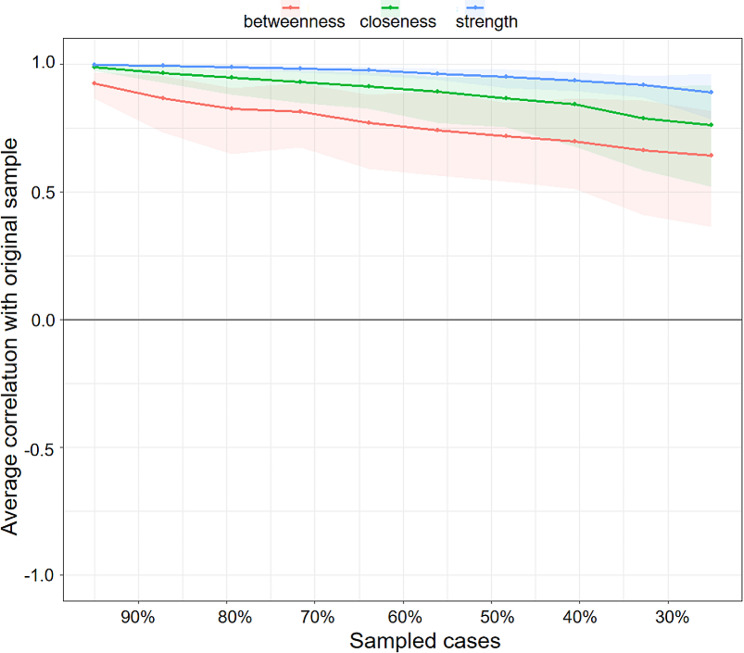
Stability of central indices. The x-axis indicates the percentage of patients in the original sample included at each step. The y-axis indicates the average correlation coefficients between the centrality indices from the original network and the centrality indices from the networks that were re-estimated after excluding increasing percentages of cases. The gray bars surrounding the colored line indicate the width of the bootstrapped CIs.

Figure 5 shows the resulting plot of the stability of centrality indices. As the percentage of the sample included in the estimates decreases (as illustrated on the X-axis, the subset samples decrease from 95% of the original sample to 25% of the sample), there is a slow decrease in the correlation between the subsample estimate and the estimate from the original entire sample. Only when the subset sample is under 30% of the original sample does the betweenness estimate fall below 0.7, but the strength and closeness estimates are still greater than 0.75.

(4) Edge weight accuracy

**Fig. 6:**
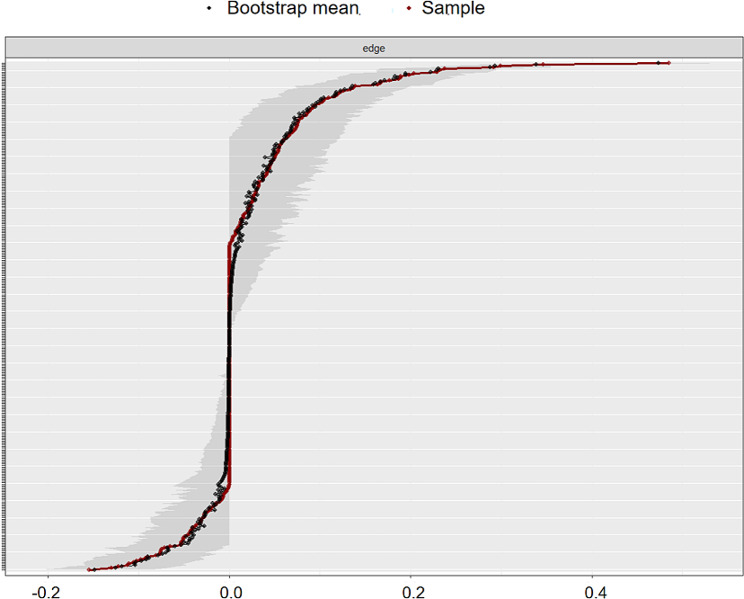
Accuracy of the edge-weight estimates (red line) and the 95% confidence intervals (gray bars) for the estimates. The x-axis represents the edges, while every line on the y-axis represents a specific edge

The bootstrapped CIs for the edge weights are shown in Fig. 6. Generally, they were relatively small, indicating that they were reasonably accurate and that many of them differed significantly from each other. Many of the edges are estimated as 0. For some edges, the estimates are larger than 0, the CIs do not include zero, or some edges are larger than 0; however, the CIs contain 0. Given the above pattern of CIs for the edge weights, the network should be interpreted with caution.

## Discussion

In this study, we constructed a network of physical symptoms, anxiety and depression symptom in older patients with advanced cancer by utilizing network analysis to examine data from a multicenter cross-sectional study in China. The results suggest that the stability of the centrality indices is reliable; notably, strength tends to be the most stable estimated centrality index in these networks, followed by closeness and betweenness. Overall, the network stability was relatively high. According to the observed network model, MDASI core symptoms and anxiety and depression symptoms were strongly correlated, with the latter two being more closely related. These findings also confirm previous findings [24, 25] that anxiety and depression have a high level of coexistence. Our study visualized the complex interaction between anxiety and depression, and the results confirmed that there is a significant relationship between the burden of physical symptoms in cancer patients and anxiety and depression [26, 27]. The majority of older cancer survivors are more likely to have additional risk factors for cancer and comorbidities [28]. The results of this study showed that 64.9% of older patients with advanced cancer had one or more symptoms, and up to 80% had anxiety and depression symptoms. These rates are higher than the overall burden of symptoms in patients with advanced cancer that our previous study revealed [14], indicating that the cancer burden of the oldest people is much greater than that of the other people and is worthy of attention. In particular, largely unaddressed comorbidities associated with cancer in China are common mental health disorders that are underrecognized and undertreated [29].

Based on network theory [7, 8], given their high centrality index scores, these symptoms may be targets for therapeutic interventions. In the whole network, ‘distress/feeling upset’ (MDASI 5) had the strongest edge connections and was the most important bridging symptom connecting different syndrome communities. Distress is common in cancer patients and survivors and may interfere with the ability to cope effectively with cancer, physical symptoms and treatment. The level of distress of patients varies according to age, sex, cancer site, treatment setting and disease progression [30]. Distress screening is recommended in guidelines and is one of the focuses of psycho-oncology. Brief screening tools can be used to identify patients who are experiencing clinically important cancer-related distress. The most widely used of these is the Distress thermometer, for which the prevalence of distress varies between 39% and 60% [31, 32]. A score of four or more on the Distress Thermometer is suggested to prompt further review of symptoms of anxiety or depression [33]. ‘Disturbed sleep’ (MDASI 4) is the most closely associated physical symptom with ‘distress/feeling upset’. The prevalence of sleep disturbance in older adults with cancer was 40%, and this symptom was associated with daily living impairment and physical activity limitations [34]. In addition, people with sleep disturbance are usually prone to comorbid anxiety and depression [35, 36]. Sleep hygiene and cognitive behavioral therapy are currently recommended for patients with sleep problems [37]. For all three centrality indices, ‘I lost interest in my appearance’ (HADS-D4) had the lowest scores. One way to understand these results is that females, who made up a larger portion of this study (71.1%), may have less awareness and concern about beauty and cosmetics [38]. Identifying the driving symptoms in the symptom cluster is an equally important question in cancer symptom management. While our network analysis based on cross-sectional data does not demonstrate causality, the centrality indices of the network provide some insights into symptom clusters. For example, we found strong direct associations between ‘drowsiness’ (MDASI 9), ‘fatigue’ (MDASI 2) and ‘shortness of breath’ (MDASI 6). The nodes are on the edges of one symptom community network in Fig. 3. If our findings are confirmed in an independent sample, future research could explore causality and evaluate interventions to help clinical workers provide individualized symptom management strategies.

Several limitations need to be considered. First, this network reveals only partial correlations and does not define causal associations. Prospective clinical studies can be conducted to verify the results of this study in the future. Second, because the participants had six various types of cancer, the impact of different cancers on the burden of symptoms was ignored; for example, lung cancer patients were more likely to have shortness of breath. Third, the strength of certain associations needs to be understood cautiously because the CIs of some edges between symptoms are large, suggesting that additional samples are needed to elucidate the strength of these associations.

## Conclusion

The symptom burden remains high in older patients with advanced cancer in China. In this study, we used network analysis to explore the associations between physical symptoms, anxiety, and depression symptoms. The visualization of interrelationships suggested that psychosomatic symptoms are often comorbid and interact with each other. “Distress/feeling restless” and “disturbed sleep” are centrally related to other symptoms; therefore, early assessment or intervention of these core symptoms may reduce the overall symptom burden. While these findings warrant verification in independent samples, this study has the potential to improve our understanding of symptom burden to provide targeted interventions to optimize symptom management in older patients with advanced cancer in China.

### Electronic supplementary material

Below is the link to the electronic supplementary material.

Supplementary Material 1

## Data Availability

The raw data supporting the conclusions of this article will be made available from the corresponding authors on reasonable request.

## Abbreviations

MDASI: MD Anderson Symptom Inventory
HADS: Hospital Anxiety and Depression Scale
HADS-A: Hospital Anxiety and Depression Scale anxiety subscale
HADS-D: Hospital Anxiety and Depression Scale depression subscale
MS: Moderate to severe
CIs: Confidence intervals
ECOG: Eastern Cooperative Oncology Group
